# Transition to family parenting in the face of the first child: a scoping review

**DOI:** 10.1590/0034-7167-2023-0487

**Published:** 2024-11-22

**Authors:** Maria Isabel Ventura Araújo, Alberto Carlos Marques Duarte, Maria Henriqueta de Jesus Silva Figueiredo, Carmen Maria da Silva Maciel Andrade

**Affiliations:** I Unidade de Saúde da Ilha de São Miguel. Ponta Delgada, São Miguel-Açores, Portugal; II Universidade dos Açores. Ponta Delgada, São Miguel-Açores, Portugal; III Escola Superior de Enfermagem do Porto. Porto, Portugal

**Keywords:** Transition, Family, Parenthood, First Child, Nursing, Transición, Familia, Paternidad, Primer Hijo, Enfermería

## Abstract

**Objectives:**

to identify and summarize the elements that characterize the family transition process in relation to the first child.

**Methods:**

a scoping review was carried out based on JBI methodology, in six databases, following the Preferred Reporting Items for Systematic reviews and Meta-Analyses extension for Scoping Reviews checklist.

**Results:**

ten articles were included with factors characterizing the transition, such as hindering/facilitating conditions that influence the process, important support structures in adaptation and strategies/responses used in the transition process.

**Final Considerations:**

elements characterizing the transition process in relation to the first child were identified. However, no theoretical explanation for this was identified. Further research should be carried out to obtain a deeper understanding of this process.

## RESUMEN

**Objetivos:**

identificar y resumir los elementos que caracterizan el proceso de transición familiar en relación al primer hijo.

**Métodos:**

se realizó una revisión de alcance con base en la metodología JBI, en seis bases de datos, siguiendo la lista de verificación *Preferred Reporting Items for Systematic reviews and Meta-Analyses extension for Scoping Reviews*.

**Resultados:**

se incluyeron diez artículos con factores que caracterizan la transición, como condiciones obstaculizadoras/facilitadoras que influyen en el proceso, estructuras de apoyo importantes en la adaptación y estrategias/respuestas utilizadas en el proceso de transición.

**Consideraciones Finales:**

se identificaron elementos que caracterizan el proceso de transición hacia el primer hijo. Sin embargo, no se ha identificado ninguna explicación teórica para esto. Será necesario realizar investigaciones adicionales para obtener una comprensión más profunda de este proceso.

## INTRODUCTION

Transition refers to the passage or movement from one state to another, characterized by flow and movement over time, involving processes and results of an adaptive interaction between the person and the environment, in the sense of reaching a new balance^(1)^.

Throughout its development, families experience different transitions, generating expected and/or unexpected changes. When a child is born, families and, in particular, parents, need to define parental roles and adapt to the new parental status. Parenting involves developing actions and interactions (physical and emotional care) that encompass children’s development and acquisition of parental identity^(2-4)^.

Transition to parenthood involves a complex set of influences and relationships between the actors involved. This complexity can have implications not only for parents’ health and well-being, but also for children’s healthy physical and emotional development, as it involves the bond/relationship between parents, adaptation to parenthood and child education^(5)^.

In this regard, parenthood is considered one of the most important and significant transitions in people’s lives, as it promotes major changes in the family and all its members, starting, in the case of a first child, a new phase in the family life cycle, also beginning to take over parental role^(6,7)^.

Although it is a normative event, the course that follows after the birth of the first child will never be the same as before, changing family identity, roles and functions^(7)^. These roles are formed based on the function of each member, according to the position they occupy in the conjugal, parental and filial subsystems. In this way, family integration allows all members to act as a group for common purposes, in order to promote their healthy development. In other words, when each member recognizes and performs specific functions and roles, understanding the limits, the family becomes a facilitator of all members’ physical and emotional health^(8-10)^.

Children act as a unifying element between generations, centralizing common goals and creating opportunities to strengthen ties. Their presence triggers a whole set of activities related to caregiving, for which parents do not yet have the skills. The instrumental and emotional support provided by grandparents usually proves to be a protective factor in adapting to parenthood^(10)^.

Transition to parenthood thus becomes a challenge for the family in terms of its ability to adapt, as well as for nursing, as it requires that nurses, together with the family, develop strategies to enhance their capabilities and resources to deal with the challenges towards a healthy transition.

However, it is not clear what information is available in the literature about what parents experience, what they do and what kind of problems and needs they feel in this transition process. Thus, knowing the elements that characterize this transition process becomes relevant for nursing care so that there is an adequate support network for families experiencing this transition process. Therefore, a scoping review was carried out to systematically map the research carried out in this area as well as to identify possible gaps in knowledge.

## OBJECTIVES

To identify and summarize the elements that characterize the family transition process in relation to the first child.

## METHODS

### Study design

A scoping review was conducted using the methodology proposed by JBI^(11)^. The choice of a scoping review was based on the main objective of this study design, such as to map evidence underlying a specific research focus, identifying gaps and constituting a preliminary effort that justifies carrying out a systematic literature review^(11)^. The protocol for this review is registered with Prospero under ID CRD42023385343.

### Methodological procedure

Considering the methodology used, and after defining the research theme, question and objectives, eligibility criteria were defined based on Participants, Concept and Context (PCC): (P) families with a first child up to two years of age; (C) focus on the transition process to parenthood; and (C) families in a cohabitation situation, seeking to answer the following question: how is transition to parenthood characterized in the family when faced with the first child?

As for study design, we considered primary quantitative, qualitative and mixed-methods studies that met the defined inclusion criteria and secondary studies such as literature reviews of different types, including meta-analyses or meta-syntheses. Studies not published in the Portuguese Open Access Scientific Repository (RCAAP - *Repositório Científico de Acesso Aberto de Portugal*) and OpenGrey database were also included.

### Data source and research strategy

Concerning the search strategy and study identification, the CINAHL Complete (via EBSCOhost), MEDLINE (via PubMed), Cochrane Central Register of Controlled Trials, Cochrane Database of Systematic Reviews, SciELO and Scopus electronic databases were used. In turn, the search for unpublished studies included RCAAP and OpenGrey. The search strategy included articles published in Portuguese, Spanish and English, as these are the languages spoken by the authors, and article selection took place between March and July 2023. There is no time limit, as no reviews on the topic were identified in an open search. Additionally, this option is consistent with the methodology used to conduct scoping reviews. The research was conducted in three stages, as recommended by the JBI manual (2020 version)^(11)^. Initially, a search was carried out in the MEDLINE (via PubMed) and CINAHL Complete (via EBSCOhost) databases, in order to identify the most used words in studies’ titles and abstracts as well as indexing terms. Subsequently, a new search was carried out separately in the CINAHL Complete (via EBSCO) and MEDLINE Complete (via PubMed) databases, using natural language search terms and indexing terms identified in the previous step, using the Boolean operators “OR” and “AND”. The identified words and terms were combined into a single search strategy, adjusted according to the specificities of each database/repository included in the review, and PCC elements, previously presented, were articulated to define the search terms and their combinations (Chart 1). Finally, a list of references of each selected study was analyzed in order to include potential additional studies.

**Chart 1:** Research strategy used in this scoping review

Search expression	Search keywords applied to titles, abstracts, topics and subject headings
1	(Family [Title/Abstract]) OR (Extended family [Title/Abstract])) OR (families [MeSH Terms])) OR (extended families [MeSH Terms])) OR (extended family [MeSH Terms]))
2	(Transition process [Title/Abstract])) OR (adaptation process [Title/Abstract])) OR (adjustment [Title/Abstract])) OR (coping skill [MeSH Terms])) OR (coping skills [MeSH Terms])) OR (coping strategies [MeSH Terms])) OR (coping strategy [MeSH Terms])) OR (adaptative behavior [MeSH Terms])) OR (adaptative behaviors [MeSH Terms]))
3	(Cohabitation [Title/Abstract])) OR (home environment [Title/Abstract])) OR (housing [MeSH Terms])) OR (home environment [MeSH Terms])) OR (home environments [MeSH Terms])
Final search expression	1 and 2 and 3

### Data extraction and analysis

Data were extracted using an extraction tool developed by the researchers in line with the objective and review question, which included author identification, year and country of origin, methodology used, information about participants and main results. This process was carried out through consensus between two reviewers. In the event of disagreements, these were resolved with the help of a third reviewer. Data synthesis was carried out in a narrative manner and through information schematization in a summary table.

After database search, all study titles and abstracts were extracted and stored using Mendeley^®^ V1.19.4 (Mendeley Ltd., Elsevier, Netherlands). Duplicate studies were eliminated. Subsequently, the articles were read and analyzed according to the titles by two reviewers and, in case of doubt or disagreement, a third reviewer intervened to make a decision. In relation to reading the abstracts and full texts, the same procedure was followed^(12,13)^.

To assess the quality of included studies, we used Critical Appraisal Tools, available on the JBI website.

Considering the objectives and research question, it was decided to summarize the main results of included studies through descriptive qualitative content analysis^(11)^. Charts 2 and 3 present the results of this review.

**Chart 2:** Summary of studies included in the review

Authors	Year/country	Methodology	Sample (N)	Study objectives	Main results
Rita Borg Xuereb; Angela Abela & Georgette Spiter^(15)^	2012 Malta	Qualitative study	13 couples	Explore the experiences of first-time parents between pregnancy and the first six months after birth.	Pregnancy is a time of preparation for the transition process; Pregnancy is characterized by being a period marked by uncertainty, disbelief and change; The postpartum period is a time of significant change in a couple’s life; The experience of caring for a child is a transformative process, a lifestyle and a responsibility that requires long-term preparation, which begins even before the pregnancy itself.
Diane Brage Hudson; Christie Campell-Grossman; Margaret Ofe Fleck; Susan M. Elek & Amy Shipman^(16)^	2003 United States of America	Quasi-experimental repeated measures study	34 couples	Test the effectiveness of an internet-based intervention, the New Fathers Network, in improving first-time parents’ self-efficacy and parental satisfaction during the first eight weeks after the birth of a baby compared with no intervention in addition to usual parenting information provided to new parents (comparison group).	The New Fathers Network, an online social support intervention, has been shown to be effective during transition to parenthood, as: Increases self-efficacy and satisfaction of first-time parents; Promotes parental autonomy and satisfaction; Provides appropriate responses to parents’ needs; Provides parents with feedback on how their parenting skills are improving and how their children’s behavior is evolving.
Cecily Young; Rachel Roberts & Lynn Ward^(17)^	2021 Australia	Qualitative study	Ten couples	Investigate couples’ memories of resilience-building experiences in the first year of parenthood.	Factors that facilitate the adaptation process were identified: Personal skills: acceptance, compassion, assertiveness and seeking help; Support structures: their partner, followed by mothers, peers and midwives; Commitment characteristics displayed by support providers: attunement to parents’ needs and good communication skills; Communication styles: respectful and the ability to actively listen and flexibly assess parents’ needs; Accessibility and punctuality: whether they were professional services or family members.
Omar Kowlessar; John R. Fox & Anja Wittkowski^(18)^	2015 United Kingdom	Qualitative study (interpretative approach)	Ten couples	Explore couples’ experiences during their first year as parents to identify their experiences and transition into parenthood.	Parents experienced feelings of despair in adapting to their new role; Pregnancy experiences, for men, symbolize the awareness of changing roles and status; Pregnancy not only affected the way men thought and felt about themselves and their social contexts, but also how their social world related to them; Fathers still seem to feel undervalued and unsupported when they seek prenatal care.
Diane Brage Hudson; Susan M. Elek & Margaret Ofe Fleck^(19)^	2001 United States of America	Mixed-methods study	44 couples	Explore parents’ experiences during their first year as parents and learn about their experiences and transition to parenthood.	Parents reported significantly lower childcare self-efficacy than mothers; Reports of mothers’ satisfaction increased over time for both mothers and fathers; At eight, 12, and 16 weeks after the baby’s birth, mothers’ and fathers’ childcare self-efficacy scores were significantly related to their degree of parental satisfaction; Parental self-efficacy regarding childcare was significantly related to satisfaction levels after 12 and 16 weeks; Parents of boys had significantly higher parental satisfaction scores than parents of girls at 12 and 16 weeks after a child’s birth.
Sonja Perren; Agnes Von Wyl; Dieter Brgin; Heidi Simoni & Kai Von Klitzing^(20)^	2005 Switzerland	Mixed-methods study	62 couples	Investigate the impact of wedding memories on marital quality (self-reports and clinical assessment) from pregnancy to 1 year after the birth of the first child.	A couple’s marital satisfaction is directly related to the quality of their dialogue; Couples’ marital satisfaction decreases one year after the birth of their child; Couples who had negative memories of their parents’ relationship reported more negative changes in the quality of their marriages; Changing roles and increased stressful experiences can challenge a young couple’s communication and conflict resolution skills; The roles that grandparents play in the life of the new family (i.e., current help and support in daily life) can affect the course of parental partnership; The couple’s memories of their families of origin predicted changes in marital quality - marital satisfaction and quality of observed dialogue - from pregnancy to one year after a child’s birth; The mental model of marriage influences both marital satisfaction and memories of the family of origin.
Susan M. Elek; Diane Brage Hudson & Carla Bouffard^(21)^	2003 United States of America	Qualitative study (longitudinal)	32 couples	Examine the effect of child gender on changes and differences in parental reports of childcare self-efficacy and marital satisfaction between 4 months and 12 months after the birth of their first child.	There are significant relationships between child care and self-efficacy, parental satisfaction, and marital satisfaction, both postpartum (four months) and at 12 months; Both mothers’ and fathers’ childcare self-efficacy increased from four to 12 months, although mothers’ scores were significantly higher than fathers’ at both time points; The sex of children did not affect childcare self-efficacy at four months after the baby was born, but by the time the child was close to 1 year old, parents of boys had significantly higher scores than parents of girls; Parental satisfaction did not change significantly from four to 12 months for either parent. However, satisfaction with the couple relationship decreased significantly from four to 12 months for both parents; The relationship between parental satisfaction and marital satisfaction suggests that the couple’s relationship may affect parents’ relationship with their children.
Kari Adamsons^(22)^	2013 United States of America	Qualitative study	55 couples	Examine predictors of relationship quality among a sample of first-time parents.	The quality of mothers’ relationships was predicted by their satisfaction with the division of child-rearing responsibilities; The quality of the couple’s relationship was related to the ideal division of parental role responsibilities.
Luciana Castoldi; Tonantzin R. Gonçalves & Rita S. Lopes^(23)^	2014 Brazil	Qualitative study	Six couples	Investigate paternal involvement throughout the first year of life of the first child, based on the psychodynamic approach of parent-baby relationships (Stern, 1997). In particular, we attempted to address the interrelations of paternal involvement with aspects such as intergenerational models of parenting, fathers’ perceptions of their own role, mothers’ perceptions of their partner as a father, the influence of the supportive matrix and paternal impressions regarding the baby’s development.	Lack of guidance, as well as mothers’ perception of their husbands’ performance as fathers, did not appear to directly influence fathers’ level of involvement with the baby; The data suggests that parents were still following traditional models of parenting, in terms of accessibility and responsibility, focusing on their role as financial provider; Parental involvement was greater in recreational activities than in childcare, for which effective models appeared to be lacking; The coexistence of identifications with modern and traditional paternity models that dynamically overlap and converge throughout transition to parenthood, implying different trajectories of paternal involvement.
Francine De Montigny; Carl Lacharité & Élyse Amyot^(24)^	2006 Canada	Mixed-methods study	160 couples	Understand the sources of support provided by fathers and mothers in the postnatal period and to examine the nature of the relationships between perceptions of social support, parental effectiveness and parental anxiety.	Social support, for these parents, did not act as a protective factor for perceptions of parental effectiveness; Nurses’ supportive practices contributed to parents’ perceptions of support as well as their perceptions of parental effectiveness; A principal components analysis revealed five dimensions of social support that accounted for 52% of the variance, such as informal support, support from a social organization, professional support, family support, and support from partners.

**Chart 3:** Transition process experience characterization

Categories	Components that represent the experience of change
Facilitating intervening conditions	Parents’ optimistic perception of the transition process to parenthood^(15,17-21,24)^.
	Preparing for parenthood in advance^(15,16,18)^.
	Timely support from healthcare professionals during pregnancy and the postpartum period, based on effective communication^(15-17,24)^.
	Personal skills and commitment strategies acquired, such as acceptance, compassion, assertiveness, help-seeking, self-care, psychological well-being and reasonable expectations^(17)^.
	Social support during the transition process, such as informal support, support from a social organization^(15-18,24)^.
	Balanced family dynamics^(17-18,20,23)^.
	Support from family of origin during pregnancy^(15)^.
	Family and friend support^(15,17,20,24)^ during pregnancy and postpartum^(15)^.
	Realization that it is the parents’ job to form an attachment to their child^(24)^.
	The use of virtual networks to promote parental self-efficacy and marital satisfaction^(16)^.
Intervening hindering conditions	Parents’ negative perception of parenting^(15)^.
	Feelings of uncertainty, disbelief and change experienced during the transition process^(15,18,20)^.
	Lack of family, friends and social support during the prenatal period^(18)^.
	Ineffective social support^(18,24)^.
Responses given during the transition process	Concern for providing quality care^(17)^.
	Use of new parenting techniques/skills after acquiring knowledge^(16-18,24)^.
	Decisions are mostly made during pregnancy^(15,18)^.
	Marital satisfaction and the quality of dialogue are highly associated^(20)^.
	The family of origin takes over a new meaning of roles during the transition process^(15,17,23)^ and influences marital quality and marital satisfaction^(20)^.
	The contrast between the different models of parenthood is experienced in a unique way by each father in terms of establishing a bond with the baby, negotiating with their intergenerational models, their partner and their support network^(18,20,23)^.
	Adoption by parents of traditional models of parenting in relation to accessibility and responsibility^(20)^, focusing on their role as financial provider^(23)^.
	Greater involvement of fathers in recreational activities than in childcare^(23)^.
Consequences in family dynamics	Emergence of new feelings and new experiences that interfere positively or negatively in family dynamics^(15-24)^.
	Awareness of the change in roles, status^(17,20)^, this being the time to plan the future as a triad^(15,18,24)^.
	Widening emotional gap between the expectation of parenthood and the pregnancy process^(18)^.
	Separation from usual social and family life upon confirmation of pregnancy^(18,20,23)^.
	Decreased marital satisfaction from pregnancy to children’s first birthday^(15,18,20,21,23,24)^.
Consequences in family dynamics	Defining new and different trajectories of paternal involvement in relation to traditional parenthood models^(23)^.
	The postnatal period is a time of crisis in a couple’s life^(15,20-22,24)^. Feelings of anxiety and uncertainty by fathers^(18)^.
	Difficulty of fathers in managing their daily lives, alternating between patterns of little involvement with their child, with periods where they more easily adhere to the “new father” pattern^(23)^.
	The roles grandparents play in the life of the new family affect the course of parental partnership^(18,20,23)^.
	Family models of marriage and parenting influence father involvement^(23)^ and couple satisfaction^(20)^.
	The entry of women into the world of work, the increase in fathers’ participation in caregiving and even the commitment to adhere to the identity of a “new father” do not erase the marks of the social and family legacy surrounding the authoritarian and provider father, especially when it comes to first-time fathers^(23)^.
	Crisis in the organization of the couple’s professional life^(15)^.
	The quality of mothers’ relationships is predicted by their satisfaction with the division of child-rearing responsibilities and is influenced by the importance that partners place on fulfilling various roles and the division of role responsibilities^(22)^.
	The relationship between parental satisfaction and marital satisfaction suggests that the couple’s relationship may affect the relationship between parents and their children^(21)^.
	Increased conflicts between the couple from pregnancy to children’s first year of life^(15,18,20,21)^.
	Fathers of boys had significantly higher levels of parental satisfaction than mothers, and although the sex of the child did not affect childcare self-efficacy at four months after the baby was born^(19)^, it increased for fathers of boys relative to fathers of girls around 1 year of age^(21)^.
	Self-efficacy in childcare: is significantly lower in fathers than in mothers; is significantly related to the couple’s parental satisfaction; increases over time and its maintenance is significantly related to parents’ satisfaction^(19)^.
	Conflicts in the couple’s life^(15,18,20,21,23,24)^.

## RESULTS

**Figure 1:**
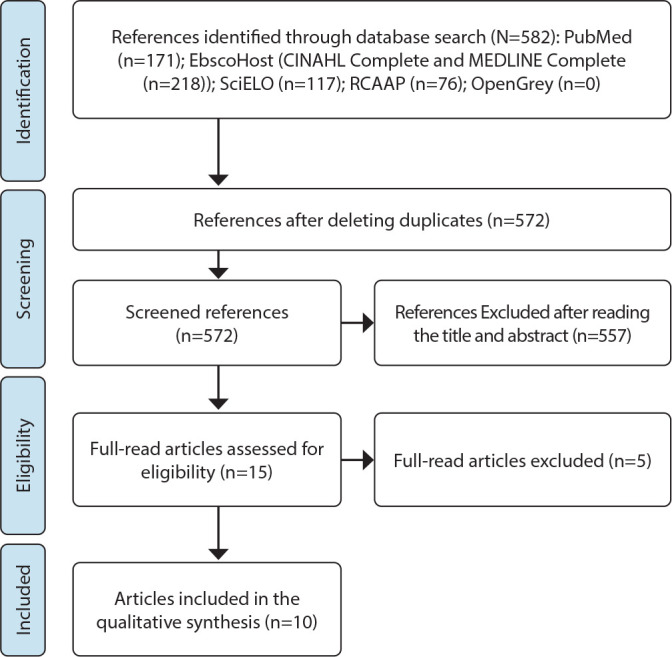
Flowchart of the study selection and inclusion process (PRISMA-ScR, 2018)

Through a search in the databases, a total of 582 studies were obtained (171 in MEDLINE via PubMed, 218 in CINAHL Complete and MEDLINE Complete, via EBSCOHost, 117 in SciELO, 76 in RCAAP and zero in OpenGrey). Ten duplicate studies were detected and removed, after which the articles were read. Through the reading and analysis of titles, 557 were excluded. After reading the full text, the 15 studies were analyzed in full, with five studies being excluded because they did not meet the inclusion criteria regarding the concept. Thus, ten studies were included in this scoping review. Figure 1 shows the flowchart of the process of study selection and inclusion according to the Preferred Reporting Items for Systematic reviews and Meta-Analyses extension for Scoping Reviews^(14)^.

Regarding the country where the studies were conducted, it was found that, of the selected articles, 40% (n=4) were conducted in the United States and America, 30% (n=3) in Europe (Malta, Switzerland and the United Kingdom), 10% (n=1) in Australia, 10% (n=1) in Brazil and 10% (n=1) in Canada. As for sample composition, all studies included families consisting of parents (couples). The results extracted from articles were those related to research question and systematic review objectives (Chart 1).

The experience of the transition process to parenthood was summarized in four categories, such as “Facilitating intervening conditions”, “Hindering intervening conditions”, “Responses given during the transition process” and “Consequences in family dynamics”, which are found in Chart 2, thus presenting the components that characterize the experience of this transition process.

## DISCUSSION

In all studies, and supporting what existing literature finds, it is recognized that transition to parenthood is characterized by being a stressful situation, full of a set of challenges and changes that can affect the family’s quality of life.

From the evidence found, it was possible to perceive that there are conditions that influence the way in which the transition process is experienced. In this regard, conditions that facilitate the process were identified, such as parents’ optimistic perception of it^(15,17-21,24)^, which helps them to face the transition moment with a different availability and acceptance. Therefore, parenthood should not be understood only as a biological process of including a newborn into the family, but a maturational process that leads to a psycho-affective restructuring that allows two adults to become parents and be able to respond to the physical, emotional and psychological needs of their children^(25)^. The fact that there is advance preparation for parenthood^(15,16,18)^, with timely support from health professionals that meets the couple’s needs^(15-17,24)^, leads the family to develop personal skills and commitment strategies, such as acceptance, compassion, assertiveness, initiative to seek help from the extended family and from the resources available to society (informal support or support from a social organization^(15-18,24)^, self-care, psychological well-being and reasonable expectations)^(17)^. Recognizing the importance of support for parenting, several theoretical perspectives support the findings, as they express the need to develop universal services and measures that promote positive parenting in the community where families live^(26,27)^. The analysis of studies therefore identified that characteristics of support engagement, whether professional services or family members, have an important impact and are quite helpful, such as communication styles and the ability to really listen and flexibly assess parents’ needs. Accessibility and punctuality were also important factors^(15-17,24)^.

It has been identified that there is a time of preparation for the event (pregnancy)^(1,2,4)^ and that taking over the role of parents is a transformative process, a lifestyle and a principle of human responsibility that requires long-term preparation. In the literature, both pregnancy, childbirth and the postpartum periods are highlighted as important moments in the process of transition to parenthood, due to marked changes they imply in the marital relationship, in daily routines of family members, or even in family and social roles^(28)^.

Another factor that contributes positively to facilitating the transition process is the coexistence of a balanced intergenerational family dynamic^(15,17,18,20,23,24)^. The family of origin thus takes over a new meaning, although there is a perception that it is the parents’ task to bond with their child, which will determine the subsequent style of relationship in the family^(24)^. For many grandparents, this is a second opportunity to be better parents. It allows them to create a new role, different from the previous one, building a new meaning, a new identity as grandparents, combined with other contextual factors, such as retirement or age^(29)^. Friends are also considered by the couple as being a favorable support for the process^(15)^.

One of the studies found also mentions that there are tools available on the internet, such as the New Fathers Network, which would be a resource for an effective internet-based social support intervention to improve first-time parents’ self-efficacy and satisfaction during transition to parenthood. The results of this pilot study support using the network to influence parents’ self-efficacy and satisfaction, since ongoing social support can provide parents with feedback that their parenting skills are improving and their efforts are appreciated^(16)^.

From the analysis of studies, conditions emerged that hinder the transition process to parenthood, which include the existence of a negative perspective of the couple towards the transition moment^(15)^ together with feelings of uncertainty and disbelief that take over a preponderant role in the way they view the entire process^(15,18,20)^. Thus, parenthood is also considered a time of crisis, due to the many changes that occur and the possibility that, during this process, parents’ capacity for self-determination, management of their needs and construction of adaptive responses may be altered, representing a risk to their health and well-being^(30)^.

Studies also suggest that lack of family support, friends and social resources in the preand post-natal periods contributes to maladaptation and/or more difficult adaptation to the phenomenon studied^(18)^. As for social resources, when they exist and are ineffective^(18,24)^, they also become an obstacle to the adaptation process. Therefore, the preand postnatal period is a critical period in which expectant parents must be involved and supported.

The birth of a baby marks the continuation of transition, not the beginning of it, with many feelings and experiences that pass from the prenatal to the postnatal period, leading the family to develop responses to the challenges that the transition process itself presents. Hence, the couple, from the moment they become parents, are concerned about providing quality care^(17)^ and are open to using new parenting techniques/skills, after acquiring knowledge^(16-18,24)^. It should be noted that the main decisions regarding parenthood are made during pregnancy^(15,18)^, and dialogue is a very important tool for this purpose. For this to happen properly, there must be balanced marital satisfaction for effective dialogue^(20)^. Transitions are, therefore, complex, multidimensional and occur due to a significant event or “turning point” that demands new patterns of response in terms of capabilities, relationships and roles^(1)^.

The results also showed that the family of origin takes over a new meaning of roles during the transition process^(15,17,23)^, influencing marriage quality and marital satisfaction^(20)^, and may be elements that contribute to a healthy development of the couple’s adaptation to the transition process, encouraging the couple to perform their new roles as parents. On the other hand, if they are not aware of their limits of action as extended family members, they become an obstacle to the healthy development of the transition process.

Thus, studies suggest that families of origin greatly influence how a couple will respond to the transition process. This is because: family models of marriage and parenting influence fathers’ involvement^(23)^ and the couple’s satisfaction^(20)^; the contrast between the different models of parenthood is experienced in a unique way by each father in terms of establishing a bond with the baby, negotiating with their intergenerational models, their partner and support network^(18,20,23)^; there are fathers who still follow traditional models of parenthood in terms of accessibility and responsibility^(20)^, focusing on their role as financial providers^(23)^; and fathers are more involved in recreational activities than in childcare^(23)^ as a result of family example. Thus, the impact of the experience of the family of origin seems to be particularly high during transition to parenthood. The new nuclear family will be the product of a couple who come from different families and cultures, bringing the marks and stories of these families. The birth of the first child is understood as the interrelation of all these stories^(31)^.

Transition to parenthood causes changes in the family, both in each member and in the family as a whole. Therefore, consequences have been identified during the transition process, which vary and even evolve depending on the family dynamics. Thus, studies have highlighted that this phase leads members to experience new feelings and new experiences that interfere positively or negatively with the family dynamics^(15-24)^. It should be noted that the positive experience of pregnancy led to awareness of the change in roles and status^(17,20)^, and this is the time to plan the future as a triad^(15,18,24)^. However, there were parents who reported that their experiences contributed to an increase in the emotional gap between the expectation of parenthood and the pregnancy process^(18)^, starting, for instance, with the fact that confirmation of pregnancy marks the beginning of the separation from the usual social and family life^(18,20,23)^. These situations can often lead to marital satisfaction decreasing from pregnancy and throughout the baby’s growth^(15,18,20,21,23,24)^. Transition to parenthood is one of the greatest changes a family system can go through. It is the moment when a husband and wife, previously a couple, become parents, the progenitors of a new family. The birth of a first child, in particular, is the couple’s first experience of parenthood^(32)^.

The studies analyzed demonstrated that the coexistence of modern and traditional parenthood models, as far as transgenerationality is concerned, dynamically overlap, conflict or converge, throughout transition and imply different trajectories of parent involvement^(23)^. There are fathers who have difficulty managing their involvement in their daily lives, alternating between patterns of little involvement with their child, with periods where they more easily adhere to the “new father” pattern^(23)^, leading to the experience of the postnatal period being experienced, sometimes, as a period of crisis between the couple^(15,20-22,24)^, and for many men, this is experienced with anxiety and uncertainty^(18)^. Thus, it can be understood that the roles that grandparents play in the life of the new family can affect the course of parental partnership^(18,20,23)^. In this regard, the meaning of being a parent is constructed over time, being a process linked to different biological, social, cultural, economic, family and personal factors^(33)^. The changes that occur in the transition process to parenthood influence male identity and family transformations will create expectations and demands in both their roles as parent and partner^(34)^.

On the other hand, the fact that women are working means that fathers are more involved in caring for the baby, which means that fathers are committed to adopting the identity of the “new father”. However, all these factors do not erase the marks of the social and family legacy surrounding the authoritarian and provider parent, especially when it comes to first-time parents^(23)^.

It should be noted that the organization of the couple’s professional life is not always an immediate consensus, and it can sometimes become a period of crisis for them^(15)^. Thus, the quality of mothers’ relationships is predicted by their satisfaction with the division of child-rearing responsibilities, and is influenced by the importance that partners place on fulfilling various roles and on the division of role responsibilities^(22)^. Furthermore, the relationship between parental satisfaction and marital satisfaction suggests that the couple’s relationship can affect parents’ relationship with their children^(21)^ and the number of conflicts increases between the couple from pregnancy and continues throughout the baby’s growth^(15,18,20,21)^. If we consider that the couple is normally seen from a relational perspective, that the transition process to parenthood occurs from pregnancy onwards and that everything changes from that moment on in the conjugal subsystem structure, the greater the interaction between fathers and children, the greater fathers’ satisfaction with their role, and this satisfaction will have a direct impact on family dynamics, namely in the relationship with their wife^(35)^.

It was also found that although mothers’ satisfaction or parenting skills were not directly related to fathers’ satisfaction or skill^(21)^ and fathers’ satisfaction did not change significantly between four and 12 months after the baby’s birth^(21)^, fathers of boys had significantly higher levels of parental satisfaction. On the other hand, the sex of the child did not affect childcare self-efficacy at four months after birth^(19)^, increasing in fathers of boys compared to fathers of girls, close to one year of age^(21)^.

It was also found that self-efficacy in childcare is significantly lower in fathers than in mothers, significantly related to the couple’s parental satisfaction, increasing over time, and its maintenance is significantly related to parents’ satisfaction^(19)^. Nowadays, parents are more aware of the importance of their presence in their children’s lives, which contributes to building stronger emotional bonds. Even so, it is still common for the fathers’ role to be seen primarily as a support for mothers. Even with changes occurring over time, it is expected that society will continue to recognize the importance of fathers’ role and functions as fundamental and significant for a balanced family dynamics^(36,37)^.

In short, the transition process to parenthood creates opportunities for disruption of routines, with the need to readapt roles creating an environment prone to relational ambiguity, with the postnatal period becoming a time of critical change in the couple’s life^(15,18,20,21,23,24)^.

### Study limitations

Of the selection of articles, 40% (n=4) were from the United States of America, 30% (n=3) from Europe (Malta, Switzerland and the United Kingdom), 10% (n=1) from Australia, 10% (n=1) from Brazil, 10% (n=1) from Canada, which may be a limitation given the differences and the importance of the unique cultural aspects of each country. This will certainly have an impact on the way families experience the transition process studied.

### Contributions to nursing

Summarizing available scientific evidence allows us to understand the elements that characterize the family transition process when faced with the first child. This summary does not allow us to draw conclusions about practice, but it can provide guidance to nurses about the transformative experience of parenthood, which can guide their clinical practice, namely: ensuring accessibility and continuity of family-centered care; establishing a partnership with the family to ensure that all their needs are met; developing empathy to support parents through dialogue, availability and relational capacity; fostering a strong, cohesive and sustained relationship of trust; valuing anticipatory care by providing parents with the knowledge necessary to better perform their parental role; and developing means that enable home visits, increasing proximity with the family.

Once the elements that characterize the family’s transition process in relation to the first child have been identified, nurses can use them to improve their intervention and help parents become aware of this stage, providing them with the skills to care for their child, in order to continue with a transition to balanced parenthood.

## FINAL CONSIDERATIONS

Understanding the transition process to parenthood contributes to developing individualized interventions to provide quality care to parents during this stage. Mapping the available evidence on the family transition process to the first child allowed not only to identify and summarize elements that characterize the transition experience but also to disseminate the available evidence. It is hoped that this scoping review will become a preliminary exercise that justifies the formulation of specific questions and the development of systematic reviews on this topic. Moreover, this review may allow identifying gaps in the literature, supporting the implementation of future primary studies.
