# Supplementary material for: Transition to family parenting in the face of the first child: a scoping review
Source: Rev Bras Enferm. 2024 Nov 22;77(5):e20230487. doi: 10.1590/0034-7167-2023-0487 (PMC11653876; doi:10.1590/0034-7167-2023-0487)
Supplement: Translation

# Translation: Transição para a parentalidade da família face ao primeiro filho: revisão de escopo

**DOI:** 10.1590/0034-7167-2023-0487pt

**Authors:** Maria Isabel Ventura Araújo, Alberto Carlos Marques Duarte, Maria Henriqueta de Jesus Silva Figueiredo, Carmen Maria da Silva Maciel Andrade

**Affiliations:** I Unidade de Saúde da Ilha de São Miguel. Ponta Delgada, São Miguel-Açores, Portugal; II Universidade dos Açores. Ponta Delgada, São Miguel-Açores, Portugal; III Escola Superior de Enfermagem do Porto. Porto, Portugal

**Keywords:** Transição, Família, Parentalidade, Primeiro Filho, Enfermagem

## Abstract

**Objetivos:**

identificar e sintetizar os elementos caracterizadores do processo de transição da família face ao primeiro filho.

**Métodos:**

efetuou-se revisão de escopo baseada na metodologia do JBI, em seis bases de dados, seguindo o *checklist Preferred Reporting Items for Systematic reviews and Meta-Analyses extension for Scoping Reviews*.

**Resultados:**

incluíram-se dez artigos com fatores caracterizadores da transição, como condições dificultadoras/facilitadoras que influenciam o processo, as estruturas de apoio importantes na adaptação e estratégias/respostas utilizadas no processo de transição.

**Considerações Finais:**

foram identificados elementos caracterizadores do processo de transição face ao primeiro filho. No entanto, não foi identificada nenhuma explicação teórica do mesmo. Investigação adicional deverá ser realizada para obter uma compreensão mais profunda desse processo.

## INTRODUÇÃO

Transição refere-se à passagem ou movimento de um estado para outro, caracterizada por fluxo e movimento ao longo do tempo, envolvendo processos e resultados de uma interação adaptativa entre a pessoa e o ambiente, no sentido de alcançar um novo equilíbrio^(1)^.

Ao longo do seu desenvolvimento, a família experiencia diferentes transições, gerando mudanças esperadas e/ou inesperadas. Aquando do nascimento de um filho, as famílias e, em particular, os progenitores necessitam definir os papéis parentais e se adaptarem ao novo estatuto parental. A parentalidade implica desenvolver ações e interações (cuidados físicos e afetivos) que compreendem o desenvolvimento da criança e a aquisição da identidade parental^(2-4)^.

A transição para a parentalidade implica uma complexidade de influências e relações entre os atores envolvidos. Essa complexidade pode ter implicações não só na saúde e bem-estar dos pais, mas também no desenvolvimento físico e emocional saudável da criança, uma vez que envolve a ligação/relação entre os progenitores, a adaptação à parentalidade e a educação da criança^(5)^.

Neste sentido, a parentalidade é considerada uma das transições mais importantes e marcantes na vida das pessoas, uma vez que promove grandes mudanças na família e em todos os seus membros, dando início, no caso de ser um primeiro filho, a uma nova fase do ciclo de vida familiar, passando, também, a assumir o papel parental^(6,7)^.

Embora seja um acontecimento normativo, o curso que se segue após o nascimento do primeiro filho nunca será o mesmo que antes, mudando a identidade, os papéis e as funções de toda a família^(7)^. Esses papéis são constituídos a partir da função de cada membro, de acordo com a posição que ocupam nos subsistemas conjugal, parental e filial. Desta forma, a integração familiar permite que todos os membros atuem, como um grupo, para fins comuns, no sentido de promover o seu desenvolvimento saudável. Em outras palavras, quando cada membro reconhece e desempenha funções e papéis específicos, compreendendo os limites, a família torna-se um facilitador da saúde física e emocional de todos os membros^(8-10)^.

O filho surge como elemento unificador entre gerações, centralizando objetivos comuns e criando oportunidades para estreitar laços. A sua presença desencadeia todo um conjunto de atividades relacionadas com os cuidados a prestar, para os quais os pais não têm, ainda, desenvolvidas competências. O apoio instrumental e emocional concedido pelos avós revela-se, habitualmente, um fator protetor da adaptação à parentalidade^(10)^.

A transição para a parentalidade torna-se, assim, um desafio à família no que diz respeito à sua capacidade de adaptação, assim como para a enfermagem, pois requer que o enfermeiro, em conjunto com a família, desenvolva estratégias de potencialização das suas capacidades e dos recursos para lidar com os desafios rumo a uma transição saudável.

Contudo, não está claro que informação está disponível na literatura sobre o que os pais vivenciam, o que fazem e que tipo de problemas e necessidades sentem nesse processo de transição. Neste sentido, conhecer os elementos caracterizadores desse processo de transição torna-se relevante para os cuidados de enfermagem, de modo que haja uma rede de apoio adequada às famílias que vivem esse processo de transição. Portanto, foi realizada uma revisão de escopo para mapear sistematicamente a pesquisa realizada nesta área, bem como para identificar eventuais lacunas no conhecimento.

## OBJETIVOS

Identificar e sintetizar os elementos caracterizadores do processo de transição da família face ao primeiro filho.

## MÉTODOS

### Tipo de estudo

Efetuou-se revisão de escopo conduzida pela metodologia proposta pelo JBI^(11)^. A escolha da revisão de escopo residiu no objetivo principal deste tipo de estudo, como mapear as evidências subjacentes a um determinado foco de investigação, identificando lacunas e constituindo um esforço preliminar que justifique a realização de uma revisão sistemática da literatura^(11)^. O protocolo desta revisão encontra-se registado na Prospero com o ID CRD42023385343.

### Procedimento metodológico

Considerando a metodologia utilizada, e após serem definidos o tema, a questão e os objetivos da pesquisa, foram definidos critérios de elegibilidade com base nos Participantes, Conceito e Contexto (PCC): (P) famílias com um primeiro filho até aos dois anos de idade; (C) foco no processo de transição para a parentalidade; e (C) famílias em situação de coabitação, procurando responder à seguinte questão: como se caracteriza a transição para a parentalidade da família face ao primeiro filho?

Relativamente ao tipo de estudo, consideraram-se estudos primários quantitativos, qualitativos e mistos que cumprissem os critérios de inclusão definidos e estudos secundários, tais como revisões da literatura de diferentes tipologias, incluindo meta-análises ou meta-sínteses. Foram também incluídos estudos não publicados no Repositório Científico de Acesso Aberto de Portugal (RCAAP) e na base de dados *OpenGrey*.

### Fonte dos dados e estratégia de pesquisa

No que concerne à estratégia de pesquisa e identificação dos estudos, foram utilizadas as bases de dados eletrônicas CINAHL *Complete* (via EBSCOhost), MEDLINE (via PubMed), *Cochrane Central Register of Controlled Trials, Cochrane Database of Systematic Reviews*, SciELO e Scopus. Por sua vez, a pesquisa de estudos não publicados incluiu o RCAAP e a *OpenGrey*. A estratégia de pesquisa compreendeu artigos publicados em português, espanhol e inglês, pois são os idiomas que os autores dominam, sendo que o período de seleção dos artigos ocorreu durante os meses de março a julho de 2023. Não apresenta limite temporal, pelo fato de, em uma pesquisa aberta, não terem sido identificadas revisões sobre o tópico. Adicionalmente, essa opção é congruente com a metodologia utilizada na realização de revisões de escopo. A pesquisa foi realizada em três etapas, tal como é recomendado pelo manual JBI (versão 2020)^(11)^. Inicialmente, foi realizada uma pesquisa nas bases de dados MEDLINE (via PubMed) e CINAHL *Complete* (via EBSCOhost), de modo a identificar as palavras mais utilizadas nos títulos e resumos dos estudos, assim como os termos de indexação. Em seguida, realizou-se uma nova pesquisa efetuada, separadamente, nas bases de dados CINAHL *Complete* (via EBSCO) e MEDLINE *Complete* (via PubMed), a partir dos termos de pesquisa em linguagem natural e dos termos de indexação identificados na etapa anterior, empregando os operadores booleanos “*OR*” e “*AND*”. As palavras e os termos identificados foram conjugados em uma estratégia de pesquisa única, ajustada de acordo com as especificidades de cada base de dados/repositório incluída na revisão, sendo que foram articulados os elementos PCC, anteriormente apresentados, para definir os termos de pesquisa e as suas combinações (Quadro 1). Para finalizar, a lista de referências de cada estudo selecionado foi analisada de modo a incluir potenciais estudos adicionais.

**Quadro 1:** Estratégia de pesquisa utilizada na revisão de escopo

Expressão de pesquisa	Palavras-chave de pesquisa aplicadas em títulos, resumos, tópicos e cabeçalhos de assuntos
1	*(Family [Title/Abstract]) OR (Extended family [Title/Abstract])) OR (families [MeSH Terms])) OR (extended families [MeSH Terms])) OR (extended family [MeSH Terms]))*
2	*(Transition process [Title/Abstract])) OR (adaptation process [Title/Abstract])) OR (adjustment [Title/Abstract])) OR (coping skill [MeSH Terms])) OR (coping skills [MeSH Terms])) OR (coping strategies [MeSH Terms])) OR (coping strategy [MeSH Terms])) OR (adaptative behavior [MeSH Terms])) OR (adaptative behaviors [MeSH Terms]))*
3	*(Cohabitation [Title/Abstract])) OR (home environment [Title/Abstract])) OR (housing [MeSH Terms])) OR (home environment [MeSH Terms])) OR (home environments [MeSH Terms])*
Expressão de pesquisa final	*1 and 2 and 3*

### Extração e análise de dados

Os dados foram extraídos com recurso ao instrumento de extração desenvolvido pelos investigadores, alinhados com o objetivo e a questão da revisão, incluindo como itens a identificação dos autores, ano e país de origem, metodologia utilizada, informação sobre os participantes e principais resultados. Esse processo foi realizado através de consenso entre dois revisores. Aquando das divergências, as mesmas foram resolvidas com recurso a um terceiro revisor. A síntese de dados foi concretizada de forma narrativa e através de esquematização da informação em uma tabela síntese.

Após realizada a pesquisa em bases de dados, todos os títulos e resumos dos estudos foram extraídos e armazenados utilizando o Mendeley^®^ V1.19.4 (Mendeley Ltd., Elsevier, *Netherlands*). Os estudos duplicados foram eliminados. Posteriormente, os artigos foram lidos e analisados segundo os títulos, por dois revisores, e, em caso de dúvida ou desacordo, interveio um terceiro revisor para a tomada de decisão. No que diz respeito à leitura dos resumos e textos integrais, procedeu-se do mesmo modo^(12,13)^.

Para avaliar a qualidade dos estudos incluídos, recorreu-se aos *Critical Appraisal Tools*, disponíveis na página da JBI.

Considerando os objetivos e a questão de pesquisa, optou-se por realizar o resumo dos principais resultados dos estudos incluídos mediante análise de conteúdo qualitativa descritiva^(11)^. Os resultados desta revisão são apresentados nos Quadros 2 e 3.

**Quadro 2:** Sumário dos estudos incluídos na revisão

Autores	Ano/ país	Metodologia	Amostra (N)	Objetivos do estudo	Principais resultados
Rita Borg Xuereb; Angela Abela & Georgette Spiter^(15)^	2012 Malta	Estudo qualitativo	13 casais	Explorar as experiências de pais pela primeira vez entre a gravidez e os primeiros seis meses após o nascimento.	A gravidez é um momento de preparação para o processo de transição; A gravidez caracteriza-se por ser um período marcado pela incerteza, descrença e mudança; O período pós-parto é um momento de mudança significante na vida do casal; A experiência de cuidar do filho é um processo transformador, um estilo de vida e uma responsabilidade que requer uma preparação de longo prazo, a qual tem início ainda antes da própria gravidez.
Diane Brage Hudson; Christie Campell-Grossman; Margaret Ofe Fleck; Susan M. Elek & Amy Shipman^(16)^	2003 Estados Unidos da América	Estudo quasi-experimental de medidas repetidas	34 casais	Testar a eficácia de uma intervenção baseada na Internet, a *New Fathers Network*, para melhorar a auto-eficácia dos pais pela primeira vez, e satisfação parental durante as primeiras oito semanas após o nascimento do bebê, em comparação com a ausência de intervenção, para além das habituais informações parentais proporcionadas aos novos pais (grupo de comparação).	A *New Fathers Network*, intervenção de apoio social *online*, mostrou-se ser eficaz durante a transição para a parentalidade, pois: Aumenta a autoeficácia e a satisfação dos pais de primeira vez; Promove a autonomia e a satisfação dos pais; Fornece respostas adequadas às necessidades dos pais; Fornece *feedback* aos pais sobre como as suas habilidades parentais estão a melhorar e como o comportamento dos seus filhos está a evoluir.
Cecily Young; Rachel Roberts & Lynn Ward^(17)^	2021 Austrália	Estudo qualitativo	Dez casais	Investigar as recordações dos casais sobre as experiências de reforço da resiliência no primeiro ano de parentalidade.	Foram identificados fatores que facilitam o processo de adaptação: Competências pessoais: aceitação, compaixão, assertividade e procura de ajuda; Estruturas de apoio: o seu parceiro, seguido pelas mães, pares e parteiras; Características de compromisso exibidas pelos prestadores de apoio: sintonia com as necessidades dos pais e boa capacidade de comunicação; Estilos de comunicação: respeitosos e a capacidade de escuta ativa e de avaliar de forma flexível as necessidades dos pais; Acessibilidade e pontualidade: fossem eles serviços profissionais ou membros da família.
Omar Kowlessar; John R. Fox & Anja Wittkowski^(18)^	2015 Reino Unido	Estudo qualitativo (abordagem interpretativa)	Dez casais	Explorar as experiências dos casais durante o seu primeiro ano como pais para captar as suas experiências e a transição para a parentalidade.	Os pais experimentaram sentimentos de desespero na adaptação ao seu novo papel; As experiências de gravidez, para os homens, simbolizam a consciência da mudança de papéis e de estatuto; A gravidez não só afetou o modo como os homens pensavam e sentiam sobre si próprios e os seus contextos sociais, mas também o seu mundo social se relacionava com eles; Os pais, homens, ainda parecem sentir-se subvalorizados e sem apoio quando recorrem ao apoio pré-natal.
Diane Brage Hudson; Susan M. Elek & Margaret Ofe Fleck^(19)^	2001 Estados Unidos da América	Estudo misto	44 casais	Explorar as experiências dos pais, durante o seu primeiro ano como pais, e conhecer as suas experiências e transição para parentalidade.	Os pais relataram autoeficácia nos cuidados infantis significativamente mais baixa do que as mães; Os relatos de satisfação das mães aumentaram ao longo do tempo para mães e pais; Às oito, 12, e 16 semanas após o nascimento do bebê, os resultados da autoeficácia dos cuidados infantis das mães e dos pais estavam significativamente relacionados com o grau da sua satisfação parental; A autoeficácia dos pais relativamente aos cuidados infantis estava significativamente relacionada com o grau de satisfação, passadas 12 e 16 semanas; Os pais de bebês do sexo masculino tinham índices de satisfação parental significativamente mais elevados do que os pais de bebês do sexo feminino às 12 e 16 semanas após o nascimento da criança.
Sonja Perren; Agnes Von Wyl; Dieter Brgin; Heidi Simoni & Kai Von Klitzing^(20)^	2005 Suíça	Estudo misto	62 casais	Investigar o impacto das recordações do casamento na qualidade matrimonial (autorrelatos e avaliação clínica) desde a gravidez até um ano após o nascimento do primeiro filho.	A satisfação conjugal do casal está diretamente relacionada à qualidade do diálogo; A satisfação conjugal do casal diminui um ano após o nascimento do filho; Os casais que tinham recordações negativas do relacionamento dos seus progenitores relataram mais mudanças negativas na qualidade dos seus casamentos; As mudanças nos papéis e um aumento das experiências estressantes podem desafiar as capacidades de comunicação e de resolução de conflitos do jovem casal; Os papéis que os avós desempenham na vida da nova família (ou seja, a ajuda atual e apoio na vida quotidiana) podem afetar o curso da parceria parental; As recordações que o casal tinha das suas famílias de origem previram mudanças na qualidade matrimonial - satisfação conjugal e qualidade do diálogo observado - desde a gravidez até um ano após o nascimento da criança; O modelo mental do casamento influencia tanto a satisfação conjugal quanto as lembranças da família de origem.
Susan M. Elek; Diane Brage Hudson & Carla Bouffard^(21)^	2003 Estados Unidos da América	Estudo qualitativo (longitudinal)	32 casais	Examinar o efeito do sexo infantil sobre alterações e diferenças entre os relatos dos pais sobre a autoeficácia dos cuidados infantis e satisfação conjugal entre quatro meses e 12 meses após o nascimento do primeiro filho.	Existem relações significativas entre os cuidados infantis e autoeficácia, satisfação dos pais e satisfação conjugal tanto no pós-parto (quatro meses) quanto aos 12 meses; A autoeficácia dos cuidados infantis, tanto das mães quanto dos pais, aumentou de quatro para 12 meses, embora as pontuações das mães fossem significativamente mais altas do que as dos pais, em ambos os momentos; O sexo da criança não afetou a autoeficácia dos cuidados infantis aos 4 meses após o nascimento do bebê, mas, na altura em que a criança tinha perto de 1 ano, os pais de rapazes tinham pontuações significativamente mais altas do que os pais de meninas; A satisfação dos pais não se alterou significativamente de quatro para 12 meses para qualquer dos pais. No entanto, a satisfação com a relação de casal diminuiu significativamente de quatro a 12 meses para ambos os pais; A relação entre a satisfação dos pais e a satisfação conjugal sugere que a relação de casal pode afetar a relação dos pais com os filhos.
Kari Adamsons^(22)^	2013 Estados Unidos da América	Estudo qualitativo	55 casais	Examinar preditores de qualidade de relacionamento entre uma amostra de pais de primeira vez.	A qualidade do relacionamento das mães era prevista pela sua satisfação com a divisão das responsabilidades na criação dos filhos; A qualidade da relação do casal foi relacionada à divisão ideal de assunção de responsabilidades de papéis pelos pais.
Luciana Castoldi; Tonantzin R. Gonçalves & Rita S. Lopes^(23)^	2014 Brasil	Estudo qualitativo	Seis casais	Investigar o envolvimento paterno ao longo do primeiro ano de vida do primeiro filho, a partir do enfoque psicodinâmico das relações pais-bebê (Stern, 1997). Em particular, tentou-se abordar as inter-relações do envolvimento paterno com aspetos como os modelos intergeracionais de parentalidade, as percepções do pai sobre o seu próprio papel, as percepções da mãe sobre o companheiro enquanto pai, a influência da matriz de apoio e das impressões paternas quanto ao desenvolvimento do bebê.	A falta de orientações, assim como a percepção das mães sobre o desempenho dos seus maridos como pais, não parecia influenciar diretamente o nível de envolvimento do pai com o bebê; Os dados sugerem que os pais ainda estavam a seguir modelos tradicionais de parentalidade em relação à acessibilidade e responsabilidade, centralizando-se no seu papel de provedor financeiro; O envolvimento dos pais era maior nas atividades recreativas do que nos cuidados infantis, para os quais pareciam faltar modelos eficazes; A coexistência de identificações com modelos de paternidade modernas e tradicionais que dinamicamente se sobrepõem, convergem, ao longo da transição, para a parentalidade, implicando diferentes trajetórias de envolvimento paterno.
Francine De Montigny; Carl Lacharité & Élyse Amyot^(24)^	2006 Canadá	Estudo misto	160 casais	Compreender as fontes de apoio promovidas pelos pais e pelas mães, em período pós-natal, e examinar a natureza das relações entre as percepções de suporte social, a eficácia parental e a ansiedade parental.	O suporte social, para esses pais, não agia como fator de proteção das percepções de eficácia parental; As práticas de auxílio das enfermeiras contribuíam para as percepções de apoio dos pais, assim como para suas percepções da eficácia parental; Uma análise dos principais componentes revelou cinco dimensões de apoio social que representam 52% da variação: apoio informal; apoio de uma organização social; apoio profissional; apoio familiar; e apoio de parceiros.

**Quadro 3:** Caracterização da experiência do processo de transição

Categorias	Componentes que representam a experiência de mudança
Condições intervenientes facilitadoras	Percepção otimista dos pais sobre o processo de transição para a parentalidade^(15,17-21,24)^.
	Preparação antecipada para a parentalidade^(15,16,18)^.
	Apoio atempado por profissionais de saúde durante a gravidez e puerpério, baseado em uma comunicação eficaz^(15-17,24)^.
	Competências pessoais e estratégias de compromisso adquiridas, como aceitação, compaixão, assertividade, procura de ajuda, autocuidado, bem-estar psicológico e expectativas razoáveis^(17)^.
	Apoio social durante o processo de transição, como apoio informal, apoio de uma organização social^(15-18,24)^.
	Dinâmica familiar equilibrada^(17,18,20,23)^.
	Apoio da família de origem durante a gravidez^(15)^.
	Apoio familiar^(15,17,20,24)^ e dos amigos durante a gravidez e puerpério^(15)^.
	Percepção de que é tarefa dos pais formar um apego ao seu filho^(24)^.
	A utilização de redes virtuais para a promoção da autoeficácia dos pais e da sua satisfação conjugal^(16)^.
Condições intervenientes dificultadoras	Percepção negativista dos pais face à parentalidade^(15)^.
	Sentimentos de incerteza, descrença e mudança vividos durante o processo de transição^(15,18,20)^.
	Falta de apoio familiar, de amigos e social no período pré-natal^(18)^.
	Apoio social não eficaz^(18,24)^.
Respostas dadas durante o processo de transição	Preocupação de prestar cuidados de qualidade^(17)^.
	Utilização de novas técnicas/competências de parentalidade após aquisição de conhecimento^(16-18,24)^.
	Decisões são tomadas, na maioria, durante o período de gravidez^(15,18)^.
	A satisfação conjugal e a qualidade do diálogo estão altamente associadas^(20)^.
	A família de origem assume um novo significado de papéis durante o processo de transição^(15,17,23)^ e influencia a qualidade matrimonial e a satisfação conjugal^(20)^.
	A contraposição dos diferentes modelos de paternidade é vivida de modo peculiar por cada pai quanto à constituição do vínculo com o bebê, à negociação com os seus modelos intergeracionais, à sua companheira e à sua rede de apoio^(18,20,23)^.
	Adoção, pelos pais, de modelos tradicionais de paternidade em relação à acessibilidade e responsabilidade^(20)^, concentrando-se no seu papel de provedor financeiro^(23)^.
	Maior envolvimento dos pais (sexo masculino) nas atividades recreativas do que nos cuidados infantis^(23)^.
Consequências na dinâmica familiar	Surgimento de novos sentimentos e novas experiências que interferem de forma positiva ou negativa na dinâmica familiar^(15-24)^.
	Consciencialização sobre a mudança de papéis, de estatuto^(17,20)^, sendo esse o tempo de planejar o futuro como uma tríade^(15,18,24)^.
	Aumento do fosso emocional entre a expetativa da parentalidade e o processo de gravidez^(18)^.
	A separação da vida social habitual e familiar aquando da confirmação da gravidez^(18,20,23)^.
Consequências na dinâmica familiar	Diminuição da satisfação conjugal desde a gravidez até um ano de idade do filho^(15,18,20,21,23,24)^.
	Definição de novas e diferentes trajetórias de envolvimento paterno em relação aos modelos de paternidade tradicionais^(23)^.
	Período pós-natal constitui um momento de crise na vida do casal^(15,20-22,24)^. Sentimentos de ansiedade e incerteza por parte dos pais (masculino)^(18)^.
	Dificuldade por parte dos pais (sexo masculino) na gestão da sua vida diária, alternando entre padrões de pouco envolvimento com o filho, com períodos onde aderem com mais facilidade ao padrão de “novo pai”^(23)^.
	Os papéis que os avós desempenham na vida da nova família afetam o curso da parceria parental^(18,20,23)^.
	Os modelos familiares de casamento e parentalidade têm influência no envolvimento do pai^(23)^ e na satisfação do casal^(20)^.
	A entrada da mulher no mundo do trabalho, o aumento da participação do pai no cuidado e mesmo o compromisso em aderir à identidade do “novo pai” não apagam as marcas do legado social e familiar em torno do pai autoritário e provedor, em especial quando se tratam de pais primíparos^(23)^.
	Crise na organização da vida profissional do casal^(15)^.
	A qualidade do relacionamento das mães é prevista pela sua satisfação com a divisão das responsabilidades na criação dos filhos e é influenciada pela importância que os parceiros dão no cumprimento dos vários papéis e a divisão de responsabilidades de papéis^(22)^.
	A relação entre a satisfação dos pais e a satisfação conjugal sugere que a relação de casal pode afetar a relação dos pais com os seus filhos^(21)^.
	Aumento de conflitos entre o casal desde a gravidez até ao primeiro ano de vida do filho^(15,18,20,21)^.
	Os pais de bebês do sexo masculino tinham índices de satisfação parental significativamente mais elevados do que as mães, e embora o sexo da criança não afetasse a autoeficácia dos cuidados infantis aos 4 meses, após o nascimento do bebê^(19)^, a mesma aumenta nos pais (sexo masculino) dos rapazes relativamente aos pais de meninas perto de 1 ano de idade^(21)^.
	A autoeficácia nos cuidados infantis: é significativamente mais baixa nos pais do que nas mães; está significativamente relacionada com a satisfação parental do casal; aumenta ao longo do tempo, e a sua manutenção está significativamente relacionada com a satisfação dos pais^(19)^.
	Conflitos na vida do casal^(15,18,20,21,23,24)^.

## RESULTADOS

Através da pesquisa nas bases de dados, obteve-se um resultado total de 582 estudos (171 na MEDLINE via PubMed, 218 na CINAHL *Complete* e MEDLINE *Complete*, via EBSCOHost, 117 na SciELO, 76 na RCAAP e zero na *OpenGrey*). Foram detetados dez estudos duplicados, os quais foram retirados, sendo que, em seguida, procedeu-se à leitura dos artigos. Através da leitura e análise dos títulos, foram excluídos 557. Após a leitura do texto completo, os 15 estudos foram analisados de modo integral, tendo sido excluídos cinco estudos por não apresentarem os critérios de inclusão referentes ao conceito. Desta forma, foram incluídos dez estudos nesta revisão de escopo. Na Figura 1, encontra-se o fluxograma do processo de seleção e inclusão dos estudos segundo o *Preferred Reporting Items for Systematic reviews and Meta-Analyses extension for Scoping Reviews* ^(14)^.

**Figura 1:** Fluxograma do processo de seleção e inclusão dos estudos (PRISMA-ScR, 2018)

No que diz respeito ao país onde foram realizados os estudos, constatou-se que, dos artigos selecionados, 40% (n=4) foram realizados nos Estados Unidos da América, 30% (n=3), na Europa (Malta, Suíça e Reino Unido), 10% (n=1), na Austrália, 10% (n=1), no Brasil, e 10% (n=1), no Canadá. Relativamente à composição da amostra, todos os estudos integraram famílias constituídas por pais e mães (casal). Os resultados extraídos dos artigos foram os relacionados à questão de pesquisa e aos objetivos da revisão sistemática (Quadro 1).

A experiência do processo de transição para a parentalidade foi resumida em quatro categorias, como “Condições intervenientes facilitadoras”, “Condições intervenientes dificultadoras”, “Respostas dadas durante o processo de transição” e “Consequências na dinâmica familiar”, que se encontra no Quadro 2, apresentando-se, deste modo, os componentes que caracterizam a experiência desse processo de transição.

## DISCUSSÃO

Em todos os estudos, e corroborando aquilo que a literatura existente constata, é reconhecido que a transição para a parentalidade se caracteriza por ser uma situação de estresse repleta de um conjunto de desafios e mudanças que podem afetar a qualidade de vida da família.

A partir da evidência encontrada, foi possível perceber que existem condições que influenciam o modo como o processo de transição é vivido. Neste sentido, foram identificadas condições que facilitam o processo, tal como a percepção otimista dos pais sobre o mesmo^(15,17-21,24)^, a qual os ajuda a encarar o momento de transição com outra disponibilidade e aceitação. Desta feita, a parentalidade não deve ser compreendida apenas como um processo biológico de inclusão de um recém-nascido à família, mas um processo maturativo que leva a uma reestruturação psicoafetiva que permite que dois adultos se tornem pais e sejam capazes de responder às necessidades físicas, afetivas e psíquicas dos seus filhos^(25)^. O fato de haver uma preparação antecipada para a parentalidade^(15,16,18)^, com um apoio dos profissionais de saúde oportuno e que vá ao encontro das necessidades do casal^(15-17,24)^, leva a que a família desenvolva competências pessoais e estratégias de compromisso, tais como aceitação, compaixão, assertividade, iniciativa para procurar ajuda, quer da família ampliada quer dos recursos que a sociedade dispõe (apoio informal ou apoio de uma organização social^(15-18,24)^, autocuidado, bem-estar psicológico e expetativas razoáveis)^(17)^. Reconhecendo a importância do apoio e suporte à parentalidade, várias perspectivas teóricas vão ao encontro dos achados, à medida que expressam a necessidade de se desenvolver serviços e medidas de caráter universal promotoras de uma parentalidade positiva na comunidade onde vivem as famílias^(26,27)^. A análise dos estudos identificou, assim, que as características de compromisso de apoios, sejam eles serviços profissionais ou membros da família, têm um impacto importante e são bastante úteis, tais como os estilos de comunicação e a capacidade de realmente ouvir e avaliar de forma flexível as necessidades dos pais. A acessibilidade e a pontualidade também foram fatores importantes^(15-17,24)^.

Foi identificado que existe um tempo de preparação para o evento (a gravidez)^(1,2,4)^, sendo que assumir o papel de pais é um processo transformador, um estilo de vida e um princípio de responsabilidade humana que requer uma preparação a longo prazo. Na literatura, tanto a gravidez quanto o parto e o pós-parto são evidenciados como momentos importantes no processo de transição para a parentalidade, pelas acentuadas mudanças que implicam quer no relacionamento conjugal, nas rotinas diárias dos membros da família, ou ainda nos papéis familiares e sociais^(28)^.

Outro fator que contribui positivamente para facilitar o processo de transição é a coexistência de uma dinâmica familiar intergeracional equilibrada^(15,17,18,20,23,24)^. A família de origem assume, assim, um novo significado, embora haja percepção de que é tarefa dos pais formar um apego ao seu filho, que irá determinar o estilo de relação subsequente na família^(24)^. Para muitos avós, essa é uma segunda oportunidade para serem melhores pais. Permite criar um novo papel, diferente do anterior, construindo um novo significado, uma nova identidade enquanto avós, aliado a outros fatores contextuais, como a reforma ou a idade^(29)^. Os amigos também são considerados pelo casal como um apoio favorável ao processo^(15)^.

Um dos estudos encontrados refere ainda que existem instrumentos na internet, tal como o *New Fathers Network*, que seria um recurso para uma intervenção eficaz de apoio social baseada na internet para melhorar a autoeficácia dos pais de primeira vez e a satisfação dos mesmos durante a transição para a parentalidade. Os resultados deste estudo piloto apoiam a utilização da rede para influenciar a autoeficácia dos pais e a sua satisfação, uma vez que o apoio social contínuo pode proporcionar aos pais *feedback* de que as suas competências parentais estão a melhorar e os seus esforços são apreciados^(16)^.

Da análise aos estudos, emergem condições que dificultam o processo de transição para a parentalidade que passam pela existência de uma perspectiva negativista do casal face ao momento de transição^(15)^ juntamente com os sentimentos de incerteza, descrença que assumem um papel preponderante na forma com encaram todo o processo^(15,18,20)^. Assim, a parentalidade é também considerada como um momento de crise, devido às muitas alterações ocorridas e à possibilidade de, no decurso deste processo, estar alterada a capacidade de autodeterminação dos pais, de gestão das suas necessidades e de construção de respostas adaptativas, representando um risco para a sua saúde e bem-estar^(30)^.

Os estudos também sugerem que a falta de apoio familiar, amigos e recursos sociais, nos períodos pré e pós-natal, contribui para uma inadaptação e/ou adaptação mais difícil ao fenômeno estudado^(18)^. Quanto aos recursos sociais, quando os mesmos existem e são ineficazes^(18,24)^, também se torna um entrave ao processo de adaptação. Assim sendo, o período pré e pós-natal é um período crítico no qual se deve envolver e apoiar os pais expectantes.

O nascimento do bebê marca a continuação da transição e não o início da mesma, com muitos sentimentos e experiências que passam do período pré-natal para o período pós-natal, levando a família a desenvolver respostas aos desafios que o próprio processo de transição apresenta. Neste sentido, o casal, a partir do momento que encara o papel de pais, tem a preocupação de prestar cuidados de qualidade^(17)^ e está aberto a utilizar novas técnicas/competências de parentalidade após aquisição de conhecimento^(16-18,24)^. É de referir que as principais decisões no que à parentalidade diz respeito são tomadas durante o período de gravidez^(15,18)^, sendo o diálogo uma ferramenta muito importante para o efeito. Para que o mesmo aconteça de forma adequada, é necessário que haja uma satisfação conjugal equilibrada para um diálogo eficaz^(20)^. As transições são, desta forma, complexas, multidimensionais e ocorrem devido a um evento significativo ou “ponto de viragem” que exige novos padrões de resposta ao nível das capacidades, relações e papéis^(1)^.

Os resultados também mostraram que a família de origem assume um novo significado de papéis durante o processo de transição^(15,17,23)^, influenciando a qualidade matrimonial e a satisfação conjugal^(20)^, sendo que podem ser elementos que contribuem para um desenvolvimento saudável da adaptação do casal ao processo de transição, encorajando o casal no desempenho dos seus novos papéis como pais. Por outro lado, se não estiverem conscientes dos seus limites de atuação como elementos da família ampliada, tornam-se um obstáculo para o desenvolvimento saudável do processo de transição.

Assim, os estudos sugerem que as famílias de origem influenciam muito a forma como o casal vai responder ao processo de transição. Isso porque: os modelos familiares de casamento e parentalidade têm influência no envolvimento do pai^(23)^ e na satisfação do casal^(20)^; a contraposição dos diferentes modelos de paternidade é vivida de modo peculiar por cada pai quanto à constituição do vínculo com o bebê, à negociação com os seus modelos intergeracionais, à sua companheira e à sua rede de apoio^(18,20,23)^; há pais que ainda seguem modelos tradicionais de paternidade em relação à acessibilidade e responsabilidade^(20)^, concentrando-se no seu papel de provedor financeiro^(23)^; e os pais (sexo masculino) envolvem-se mais nas atividades recreativas do que nos cuidados infantis^(23)^, fruto de exemplo familiar. Desta feita, o impacto da experiência da família de origem parece ser particularmente elevado durante a transição para a parentalidade. A nova família nuclear será a produção de um casal que vem de diferentes famílias e culturas, trazendo as marcas e as histórias dessas famílias. O nascimento do primeiro filho é entendido como a inter-relação de todas essas histórias^(31)^.

O processo de transição para a parentalidade provoca na família alterações, quer em cada elemento quer na família no seu todo. Neste sentido, foram identificadas consequências no decorrer do processo de transição, as quais variam e até evoluem consoante com a dinâmica familiar. Deste modo, os estudos destacaram que essa fase leva os membros a vivenciarem novos sentimentos e novas experiências que interferem de forma positiva ou negativa na dinâmica familiar^(15-24)^. É de referir que a experiência positiva da gravidez levou à consciência da mudança de papéis e de estatuto^(17,20)^, sendo este o tempo de planejar o futuro como uma tríade^(15,18,24)^. No entanto, houve pais que referiam que as experiências vividas contribuíram para um aumento do fosso emocional entre a expetativa da parentalidade e o processo de gravidez^(18)^, a começar, por exemplo, no fato de a confirmação da gravidez marcar o início da separação da vida social habitual e familiar^(18,20,23)^. Essas situações podem levar, muitas vezes, a que a satisfação conjugal vá diminuindo desde a gravidez e ao longo do crescimento do bebê^(15,18,20,21,23,24)^. A transição para a parentalidade é uma das maiores mudanças por que o sistema familiar pode passar. É o momento em que o marido e a mulher, anteriormente um casal, se tornam pais, progenitores de uma nova família. O nascimento do primeiro filho, em especial, é a primeira experiência de parentalidade vivida pelo casal^(32)^.

Os estudos analisados demonstraram que a coexistência de modelos de paternidade modernos e tradicionais, no que se refere à transgeracionalidade, se sobrepõem, conflitam ou convergem dinamicamente, ao longo da transição e implicam diferentes trajetórias de envolvimento paterno^(23)^. Há pais que manifestam dificuldade em gerir o envolvimento na sua vida diária, alternando entre padrões de pouco envolvimento com o filho, com períodos onde aderem com mais facilidade ao padrão do “novo pai”^(23)^, para que a experiência do período pós-natal seja vivida, algumas vezes, como um período de crise entre o casal^(15,20-22,24)^, sendo que, para muitos homens, esta é experimentada com ansiedade e incerteza^(18)^. Assim, pode-se entender que os papéis que os avós desempenham na vida da nova família podem afetar o curso da parceria parental^(18,20,23)^. Neste sentido, o significado de ser pai vai, ao longo do tempo, construindo-se, sendo um processo ligado a diferentes fatores biológicos, sociais, culturais, econômicos, familiares e pessoais^(33)^. As alterações que ocorrem no processo de transição para a paternidade influenciam a identidade masculina, e as transformações familiares irão criar expectativas e exigências tanto nas suas funções de pai quanto nas de parceiro^(34)^.

Por outro lado, o fato de as mulheres terem a sua atividade laboral leva a crer que haja um aumento da participação do pai no cuidado ao bebê, havendo, deste modo, o compromisso, por parte do pai, em aderir à identidade do “novo pai”. No entanto, todos esses fatores não apagam as marcas do legado social e familiar em torno do pai autoritário e provedor, em especial quando se tratam de pais primíparos^(23)^.

É de referir que nem sempre a organização da vida profissional do casal é de consenso imediato, podendo, às vezes, se tornar um período de crise para eles^(15)^. Desta feita, a qualidade do relacionamento das mães é prevista pela sua satisfação com a divisão das responsabilidades na criação dos filhos, e é influenciada pela importância que os parceiros dão no cumprimento dos vários papéis e na divisão de responsabilidades de papéis^(22)^. Além disso, a relação entre a satisfação dos pais e a satisfação conjugal sugere que a relação de casal pode afetar a relação dos pais com os seus filhos^(21)^, e o número de conflitos aumenta entre o casal desde a gravidez e perdura durante o crescimento do bebê^(15,18,20,21)^. Se considerar que o casal é normalmente visto em uma perspectiva relacional, que o processo de transição para a parentalidade acontece a partir da gravidez e tudo se altera a partir desse momento na estrutura do subsistema conjugal, quanto maior for a interação entre pais e filhos, maior é a satisfação do pai com o seu papel, e essa satisfação repercutir-se-á diretamente na dinâmica familiar, nomeadamente no relacionamento com a sua mulher^(35)^.

Também verificou-se que, embora a satisfação ou as competências parentais das mães não se relacionassem diretamente com a satisfação ou aptidão dos pais^(21)^ e a satisfação dos pais não se tenha alterado significativamente entre os quatro e os 12 meses após o nascimento do bebê^(21)^, os pais (sexo masculino) de bebês do sexo masculino têm índices de satisfação parental significativamente mais elevados. Por outro lado, o sexo da criança não afetou a autoeficácia dos cuidados infantis, aos quatro meses após o nascimento^(19)^, aumentando nos pais (sexo masculino) dos rapazes relativamente aos pais de meninas perto de 1 ano de idade^(21)^.

Constatou-se também que a autoeficácia nos cuidados infantis é significativamente mais baixa nos pais do que nas mães, estando significativamente relacionada à satisfação parental do casal e aumentando ao longo do tempo, e a sua manutenção está significativamente relacionada à satisfação dos pais^(19)^. Hoje em dia, os pais estão mais conscientes da importância da sua presença na vida dos filhos, o que contribui para a construção de laços afetivos mais fortes. Mesmo assim, ainda é comum que o papel do pai seja visto principalmente como um apoio à mãe. Mesmo com mudanças a acontecer ao longo do tempo, é esperado que a sociedade continue reconhecendo a importância do papel e funções dos pais como fundamentais e significativas para uma dinâmica familiar equilibrada^(36,37)^.

Em suma, o processo de transição para a parentalidade cria oportunidades para a disrupção de rotinas, com a necessidade de readaptação de papéis, criando um ambiente propenso à ambiguidade relacional, sendo que o período pós-natal torna-se um tempo de mudança crítica na vida do casal^(15,18,20,21,23,24)^.

### Limitações do estudo

Da seleção de artigos, 40% (n=4) eram dos Estados Unidos da América, 30% (n=3), da Europa (Malta, Suíça e Reino Unido), 10% (n=1), da Austrália, 10% (n=1), do Brasil, 10% (n=1), Canadá, o que pode ser uma limitação do estudo, devido às diferenças e à importância dos aspetos culturais únicos de cada país. Isso certamente terá impacto na forma como as famílias vivenciam o processo de transição estudado.

### Contribuições para a área da enfermagem

Sintetizar a evidência científica disponível permite-nos conhecer os elementos caracterizadores do processo de transição da família face ao primeiro filho. Este resumo não permite tirar conclusões sobre a prática, mas pode deixar orientações aos enfermeiros sobre a transformadora experiência da parentalidade, podendo direcionar a sua prática clínica, nomeadamente: assegurar a acessibilidade e continuidade dos cuidados centrados na família; estabelecer uma parceria com a família para assegurar que todas as suas necessidades sejam acompanhadas; desenvolver empatia para apoiar os pais através do diálogo, disponibilidade e capacidade relacional, potenciando uma forte, coesa e sustentada relação de confiança; valorizar os cuidados antecipatórios, facultando aos pais os conhecimentos necessários ao melhor desempenho da sua função parental; e desenvolver os meios que possibilitem a visitação domiciliária, aumentado a proximidade com a família.

Estando os elementos caracterizadores do processo de transição da família face ao primeiro filho identificados, os enfermeiros poderão utilizá-los para melhorar a sua intervenção e ajudar os pais na consciencialização dessa etapa, possuindo os mesmos de competências para cuidar do seu filho, de forma a prosseguirem com uma transição para a parentalidade equilibrada.

## CONSIDERAÇÕES FINAIS

Conhecer o processo de transição para a parentalidade constitui um contributo para o desenvolvimento de intervenções individualizadas, de modo a prestar cuidados de qualidade aos pais durante essa etapa. O mapeamento da evidência disponível sobre o processo de transição da família face ao primeiro filho permitiu não só identificar e resumir elementos caracterizadores da experiência de transição, mas também disseminar a evidência disponível, esperando-se que esta revisão de escopo se torne um exercício preliminar que justifique a formulação de questões específicas e desenvolvimento de revisões sistemáticas relativamente a esse tema. Adicionalmente, esta revisão poderá permitir identificar lacunas na literatura, sustentando a realização de futuros estudos primários.
